# Editorial: Regeneration from cells to limbs: past, present, and future

**DOI:** 10.3389/fcell.2023.1229613

**Published:** 2023-06-14

**Authors:** Pamela Imperadore, Kathryn Maxson Jones, Jennifer R. Morgan, Fabio De Sio, Frank W. Stahnisch

**Affiliations:** ^1^ Department of Biology and Evolution of Marine Organisms, Stazione Zoologica Anton Dohrn, Napoli, Italy; ^2^ Association for Cephalopod Research—CephRes, Napoli, Italy; ^3^ Center for Medical Ethics and Health Policy, Baylor College of Medicine, Houston, TX, United States; ^4^ Department of History, Purdue University, West Lafayette, IN, United States; ^5^ The Eugene Bell Center for Regenerative Biology and Tissue Engineering, Marine Biological Laboratory, Woods Hole, MA, United States; ^6^ Institut für Geschichte, Theorie und Ethik der Medizin, Centre for Life and Society, Medizinische Fakultät, Heinrich Heine Universität Düsseldorf, Düsseldorf, Germany; ^7^ Alberta Medical Foundation/Hannah Professor in the History of Medicine and Health Care, University of Calgary, Calgary, AB, Canada

**Keywords:** experimental biology, history and philosophy of science, model organisms, regeneration, epistemology, evolutionary novelties, emerging model organisms

Since the early 20th century, scientific interest in regeneration has steadily increased, fueled by hopes of applying basic knowledge of regeneration in complex living systems to clinical problems. Yet, partly because of the inherent complexity of the concept itself -- which covers everything from structural repair in unicellular forms to functional restitution of organs and appendices -- and partly as a consequence of historical contingencies in the development of the field, limited success has been achieved thus far in developing a unified framework for interpreting regeneration. Voluminous, world-class research on various aspects is ongoing, yet organizing a cohesive, interdisciplinary research community centered on regeneration is also an outstanding challenge, as evidenced by the fact that, at present, no dedicated journal for reporting research on animal regeneration even exists. Thus, the Editors welcomed the venue of *Frontiers in Cell and Developmental Biology* for this Research Topic, which offered a platform on which contributions from experimental biologists could meet those from historians and philosophers of science concerned with the epistemological aspects and sociocultural contexts of experimental work. The impetus for this way of thinking was a challenge from the then-President of the James S. McDonnell Foundation, Dr. Susan Fitzpatrick., who in 2019 asked the leaders of several working groups at the Marine Biological Laboratory (MBL) in Woods Hole, Massachusetts to “think differently” about regeneration: for instance, at various biological levels, across the animal kingdom, and in its philosophical and historical dimensions^1^. This challenge eventually led to the idea of bringing together research papers exploring regeneration along these intersecting lines. A defining feature of some of the papers in this Research Topic, therefore, is direct collaborations between biologists, historians, and philosophers of science, working together to provide wider and deeper perspectives on the multiplicity of animal models for studying regeneration, research questions in regenerative biology, and the contexts and changes through time that have been associated with these models and research programs.

The two Perspective articles in this Research Topic (MacCord and Maienschein, Fitzpatrick et al.) clearly show the breadth and complexity of the issue. Through historical examples, MacCord and Maienschein provide an overview of the epistemological changes that have characterized research on regeneration as a biological phenomenon since the 19th century, noting (for instance) the early emphasis on whole, complex systems and comparative perspectives, the shift towards model organisms and a molecular-mechanical approach in the 20th century, and different attempts at translating biological results into practice, which have met with varying degrees of success. Focusing on the example of hand transplantation and associated prosthetics and recovery of function, Fitzpatrick et al. then explore the biological, clinical, social, and ethical dimensions of different, sometimes competing, and converging therapeutic strategies. Building on the two Perspectives, the further 25 articles (12 reviews and 13 original research articles, representing the work of over 100 authors) address the study of regeneration from cells to complex structures in numerous organisms, spanning from protists, such as *Stentor coeruleus* (Marshall), to mammals (e.g., Suarez-Berumen et al.) (Figure 1). The range of species represented includes those for which sequenced genomes and molecular tools have long been available, and which therefore carry the label of traditional “model organisms” (Ankeny and Leonelli, 2021), and several less conventional experimental systems for which new opportunities are emerging, thanks to tools such as genome editing.

**FIGURE 1:**
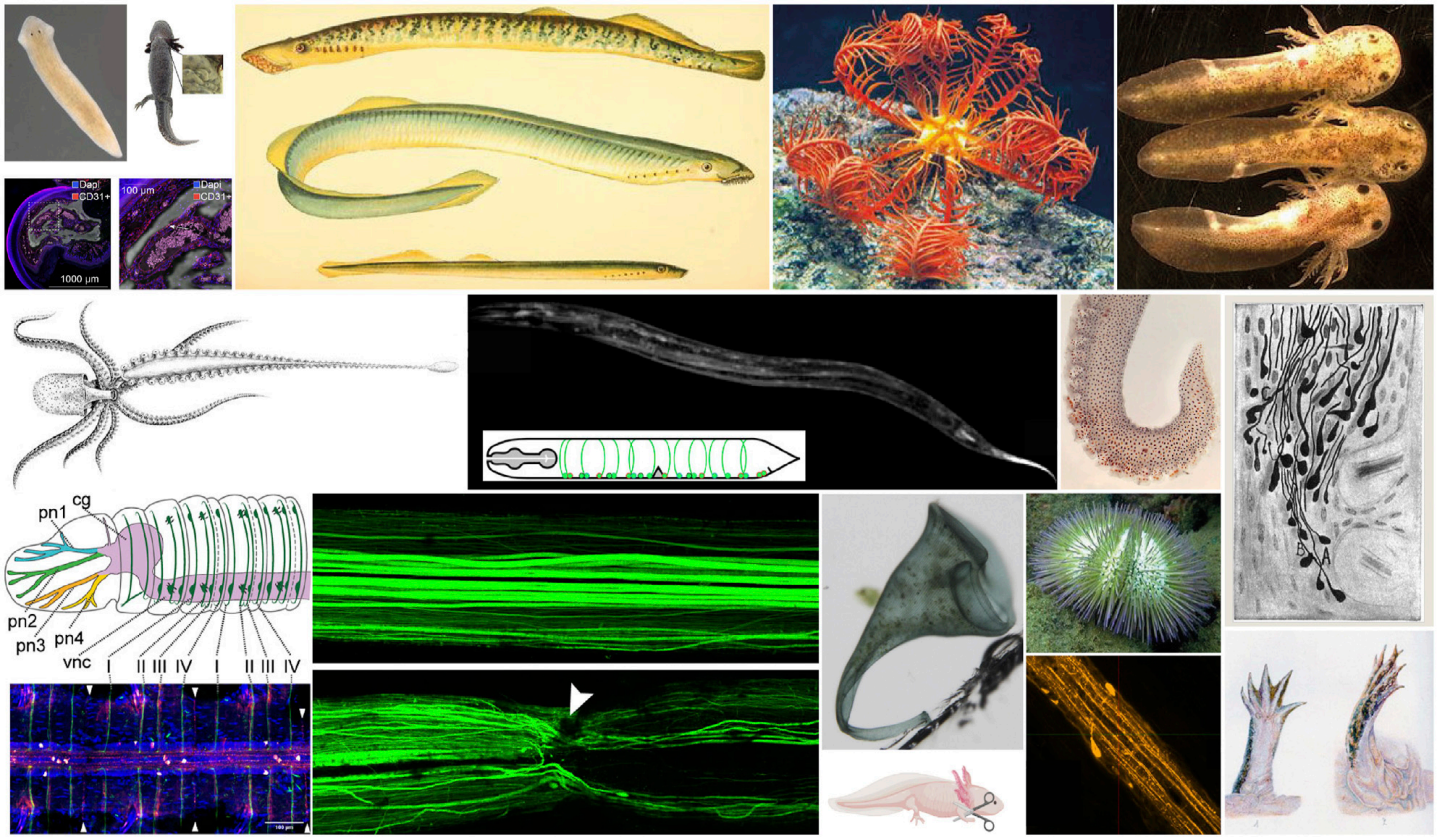
Range of organisms represented in this Research Topic, both from HPS and biological contributions. Figures adapted from: Almazan et al., Lovely et al., Hoffseth et al., Maxson Jones and Morgan, Medina-Feliciano and García-Arrarás, Voss et al., De Sio and Imperadore, Harreguy et al., Imperadore et al., Stahnisch, Martinez Acosta et al., Marshall, Debuque et al., Reiß. Images used with permission as stated under Creative Commons license: https://creativecommons.org/licenses/by/4.0/.

Unconventional models and their associated genomic and/or evolutionary novelties are recurring themes in this Research Topic, which also have enriched the field overall with a large variety of organisms and approaches. The exclusive use of traditional model systems, indeed, has for at least three decades been questioned by biologists as well as historians and philosophers of science, and the possibility of applying cutting-edge technologies to less well-studied, regeneration-competent species is increasing our chances of success in uncovering both common pathways and alternative regenerative strategies (Alvarado, 2004; Alvarado et al., 2018, De Sio and Imperadore). For instance, *S. coeruleus*, the giant heterotrichous ciliate protist, offers an impressive example of single-cell regenerator, one that is able to constantly re-establish correct patterning after any kind of disturbance. In this Research Topic, it is proposed as a model to investigate the origins of cellular geometry and single-cell repair, in order to shed light on animal development and regeneration more generally (Marshall). Echinoderms, among deuterostomes, also have allowed for the identification of conserved molecules and pathways, as well as a great number of orphan genes (unknown genes with no significant homology in any other species) active during spine, pedicellariae, arm, viscera, and pyloric caeca regeneration, thanks to high-throughput methods and the introduction of functional studies (Medina-Feliciano and García-Arrarás). Moreover, lampreys and goldfish permit detailed studies of neural regeneration and have led to the discovery that recovery of locomotor behaviors can occur despite imperfect axon regeneration, due to compensatory neural plasticity (Zottoli et al., Maxson Jones and Morgan).

Regeneration studies also provide novel educational opportunities, as Martinez Acosta et al. note, proposing *Lumbriculus* worms as “accessible models for the Lab and the Classroom.” Used since the mid-18th century to investigate regeneration, these worms are easy to care for and culture. They also are practically inexpensive, and they are recently opening to the ‘-omics’ era. Considering the availability of regenerating and non-regenerating worms, as well as of species endowed with anterior and/or posterior regeneration and indeterminate growth (Ribeiro et al., 2018; Ribeiro et al., 2019), the annelids offer excellent models for answering many outstanding biological and biomedical questions related to regeneration, evo-devo, physiology, and ecology.

Novel microscopic imaging techniques, applied to the study of regenerative phenomena, also are proving advantageous in studies of several emerging experimental species. Indeed, the scarcity of commercially available markers experienced by researchers working with non-traditional model organisms has represented a limit to their use until recently. Nevertheless, label-free multiphoton microscopy, as applied to the regenerating arm of *Octopus vulgaris*, has provided fundamental morpho-chemical information that appears promising for its use in a species-independent way (Imperadore et al.).

These few cases are enlightening and allow us to envision how the increasing use of emerging systems can guide in tackling fundamental, unresolved questions, expanding our knowledge of exceptionally complex biological phenomena.

In different ways, the history and philosophy of science (HPS) contributions in this Research Topic also accepted the challenge that MacCord and Maienschein presented in their Perspective: making history and philosophy of biology relevant to biology itself, for instance by identifying assumptions in past research to clarify limitations of and new opportunities for the present. Barbara and Stahnisch both underscore contextualized shifts in the meaning of regeneration, which has depended in large measure on the types of tissues that have been studied (Barbara) and the “thought styles” and experimental approaches of different scientific communities (Stahnisch). Barbara notes how the concept of regeneration has shifted in meaning since Antiquity in studies of soft tissue and peripheral nerve, and Stahnisch focuses on the specificities of studies of brain “plasticity” in biology and neurology since the 19th century. In addition, in his study of Rhoda Erdmann (1870–1935) and the development of tissue culture -- a method that has served regeneration scholars and many other biologists -- Fangerau emphasizes the social/communitarian dimensions of experimental biology, highlighting tissue culture research as “an academic niche for underprivileged scientists,” including women.

Moreover, the contributions examining individual species, from both the HPS and biological perspectives, raise complementary questions, investigating the historical and epistemic rationales for strategic choices of models. The lamprey (Hu et al., Maxson Jones and Morgan) has long proven to be a productive choice of organism for studying anatomical and molecular features of CNS regeneration conserved through the vertebrate lineage, while cephalopods (De Sio and Imperadore, Imperadore et al.) and echinoderms (Medina-Feliciano and García-Arrarás) have helped biologists investigate diversity, meaning “the novel strategies different taxa evolved to promote regeneration of tissues and organs”. Reiß, in addition, has shown how the axolotl’s remarkable regeneration capacities raised the latter to the status of a *bona fide* Research Topic in the 20th century, after the organism first gained cache in biology in the contexts of metamorphosis and experimental zoology. Salamanders, indeed, and the Mexican axolotl (*Ambystoma mexicanum*) in particular, are nowadays established and axiomatic organisms for the study of regeneration (Joven et al., 2019), a status confirmed by the number of contributions included in this Research Topic. Salamanders prove particularly useful for investigating the involvement and role of conserved pathways in limb development and regeneration (Lovely et al., Wells et al.), the epigenetic control of transcriptional regulation in tail regeneration (Voss et al.), and the contributions of pro-regenerative, liver-derived macrophages in limb repair (Debuque et al.). Similarly, the highly-regenerative planarians are well represented in this Research Topic, revealing their power in studies of mechanisms of regeneration across scales, from molecules to behavior (Almazan et al., Allen et al.).

Despite the contributions offered by non-conventional organisms, however, methodological challenges still remain, particularly related to transgenic approaches for functional studies. Thus, while model organisms, in the traditional sense, are sometimes endowed with limited regenerative abilities, they nonetheless contribute to the advancement of the field, as the articles in this Research Topic examining *Mus musculus* (Hoffseth et al., Suarez-Berumen et al.), *Caenorhabditis elegans* (Harreguy et al.), and *Xenopus laevis* (Ivanova et al.) demonstrate. Indeed, through transgenic animals, overexpression experiments, long-lasting cell culture, and other molecular methods, these species continue to offer biologists unique opportunities for in-depth investigations of regenerative phenomena, in ways that emerging models are only just attempting to pursue.

Collectively, the diversity of species now involved in regenerative studies -- including both traditional and emerging model organisms -- sustains a comparative approach, which can highlight shared features, molecules, and mechanisms involved in various biological systems. Katz et al., for instance, identify ATF3 as a common neural pro-regenerative transcription factor in vertebrates with a high degree of sequence homology across phyla, confirming it as one of the most actively induced genes in highly regenerative species following CNS damage (e.g., spinal cord injury (SCI) in zebrafish and SCI and brain injury in lamprey) that also is actively induced in mammals (rodents and human cell lines) after injury in several tissues. Similarly, Avalos and Forsthoefel propose cell-cell signaling through extracellular vesicles (EVs) and their cargos as ubiquitous mechanisms occurring in all systems, both in physiological turnover as well as in injury repair. EVs, indeed, can transport cargo that regulates apoptosis, cell survival, and tissue growth, as well as micro-RNAs (miRNAs), many of which have already been demonstrated to play active roles in regeneration across distinct phyla. Interestingly, despite their discovery in the early 1990s, miRNAs have been proposed recently as new and potentially powerful targets for therapeutic intervention against various pathological conditions, including SCI in humans (as reviewed in Boido and Vercelli). In their mini-review, Boido and Vercelli suggest combined therapeutic approaches with the aims of activating transcriptional cascades to promote axonal regrowth, restoring damaged neuronal circuitries, and reverting the inhibitory mechanisms occurring in the mammalian CNS after lesions that generate a hostile environment for regeneration.

Taken together, the contributions to this Research Topic hint at further agendas: both for HPS and biological scholars and for potential areas of fruitful collaboration. Despite significant existing studies (e.g., Dinsmore, 2007; Stahnisch, 2016; Stahnisch et al., 2019), historical changes in the meanings and contexts of the concept itself are still under-investigated, and thus they hold the promise of providing fresh views on the interactions of bio-medical research and public health priorities and on ethical considerations of the limits of medicine.

Finally, the varied landscape of research options—in terms of techniques, animal models, approaches, and objectives—collectively painted by the contributions to this Research Topic calls for a difficult, but promising, common endeavor as the future of regeneration research unfolds. Truly comparative regeneration studies are still greatly needed, both in order to establish which molecular pathways and strategies are most viable to rebuild and replace lost structures and functions, and to move toward clinical applications. However, various factors make comparing regeneration across species extremely challenging, including fundamental differences in the species themselves as well as in the injury models employed (i.e., in their tissue composition and time courses for regeneration, to name but a few variables). Thus, what we need now is a new vision for how to unify these experimental variables across species, a challenge requiring interdisciplinary perspectives. Major funding sources also are needed to support this work. Indeed, the high ratio of emerging/unconventional models to traditional model organisms presented here may be a skewed and partial picture, yet it resonates with many old and new cries for revising the balance amongst model organisms, traditional “translational” approaches focused on higher vertebrates and mammals, and more “biological” perspectives, harkening back to the 19th century and earlier and focused on a multiplicity of species and various dimensions of comparisons between them (e.g., Alvarado, 2004; Alvarado et al., 2018). Moving forward, realizing the promises of regenerative medicine, and maximizing the applications of the research that already has taken place, may well require a reevaluation of the meaning of comparative research in light of molecular approaches, not to mention a radical reassessment of the very concept of “translation”. For example, in what ways does biological research gain relevance to medicine? How is this “relevance” defined? Or “promise?” “Or even the term “regeneration” itself?” There surely are many illuminating solutions emerging in each of the fields here represented. But such complex and cogent questions call for novel, courageous, and collective efforts to eschew tunnel vision.

## Footnotes

1
https://mcdonnellinitiativeatmbl.com/. Accessed 26 April 2023.
